# Executive Function Training for Deaf Children: Impact of a Music Intervention

**DOI:** 10.1093/deafed/enab026

**Published:** 2021-09-02

**Authors:** Kathryn Mason, Chloe Ruth Marshall, Gary Morgan

**Affiliations:** The Deafness Cognition and Language (DCAL) Research Centre, University College London (UCL); UCL Institute of Education, University College London (UCL); City, University of London

## Abstract

Several studies have reported poor executive function (EF) development in deaf children with subsequent impacts on their social and academic attainment. This paper describes the results of a music-based EF intervention designed for deaf children and carried out in two sets of primary schools. This is the first classroom-based EF training study with deaf children, and it also incorporates a replication phase. The intervention was a within-subject crossover design, with 29 deaf children aged 7–11 years who participated in both an EF and an art class control activity, each lasting 10 hours over 5 weeks. Non-verbal EF skills were assessed at pre-test, the crossover point, and post-test. Findings indicated that the EF intervention led to an improvement in participants’ working memory and inhibitory skills in comparison with their performance on the same tasks after the control activity. The findings were not uniform for all EFs targeted nor for all cognitive ability levels in the sample. We discuss the implications of our findings for deaf children with different ability levels and for how EF interventions can be further improved.

Executive functions (EF) are a complex set of cognitive abilities, which enable us to coordinate mental processes, manipulate information, solve novel problems, sequence information, and generate new strategies to accomplish goals in a flexible way (Diamond, 2012; Henry, Messer, & Nash, 2012; Miyake & Friedman, 2012). In addition to storing information in short-term memory, children need to be able to process information flexibly, inhibit non-useful responses, and manage the input in order to achieve success on higher-level cognitive tasks. Much EF research focuses on three core areas: inhibition; flexibility and working memory which some suggest underpin other more complex EFs such as planning and cognitive fluency (Friedman & Miyake, 2017).

Deaf children, like some other groups (e.g., Autism: Demetriou et al., 2018; language impairment: Henry et al., 2012), are at risk of delayed development of EF, particularly in working memory and inhibition (Beer, Kronenberger, & Pisoni, 2011; Burkholder & Pisoni, 2003; Figueras, Edwards, & Langdon, 2008; Hintermair, 2013). There is still debate as to whether this delay is a direct result of deafness, or due to other factors affecting cognitive development, especially a delay in language development (Botting et al., 2017; Figueras et al., 2008; Hall, Eigsti, Bortfeld, & Lillo-Martin, 2016; Jones et al., 2020; Kronenberger & Pisoni, 2020; Morgan & Dye, 2020; Pisoni & Cleary, 2003).

The fact that early experiences can lead to differential outcomes in EF skills suggests that development is sensitive to environmental factors. Consequently, research attention has focused on how to enhance EF skills in different populations, including via computerized training (Klingberg et al., 2005), aerobic exercise (Hillman, Erickson, & Kramer, 2008), martial arts and mindfulness (Flook et al., 2010), and classroom curricula such as Montessori (Lillard et al., 2017) and tools of the Mind (Bodrova & Leong, 2007). These diverse activities have been found to have a positive impact on children and adults’ EF skills with varying levels of effect size.

During the design and implementation of EF interventions, previous research has shown that EF tasks, which fall within the child’s level of potential development are the most effective at improving EF skill. In contrast, tasks that the child can complete with ease do not train or develop EFs (Diamond, 2012). Successful EF interventions also contain an element of repetition and practice, which enable children to strengthen and develop their skills (Klingberg et al., 2005). A final characteristic of a successful intervention is that the participants are engaged and motivated to take part in the activity (Diamond, 2012). It is important to ensure that an activity has the potential to become more challenging as children improve, in order to keep them engaged and motivated. Relatively, few research studies have looked at “how much” training is required to impact on a child’s EF skills, but it is generally accepted that the longer a child is engaged in an intervention program, the more likely it is that EF improvements will be seen.

Music programs have been studied with a view to them potentially enhancing EF (Bowmer, Mason, Knight, & Welch, 2018; Habibi, Damasio, Ilari, Elliott Sachs, & Damasio, 2018; Moreno et al., 2011; Williams, 2018). Correlational and intervention studies of hearing children undergoing music training consistently show that they perform better in fine-motor skill, rhythm perception, and auditory discrimination (Besson, Schön, Moreno, Santos, & Magne, 2007; Costa-Giomi, 2005; Slater et al., 2015). The playing of music is highly complex and includes repetition and practice, and strong motivational and emotional rewards (Diamond, 2012). A review by Benz, Sellaro, Hommel, and Colzato (2016) reports that musical training can lead to far-transfer effects in domains such as verbal intelligence and EF. In a recent meta-analysis of music training studies, Sala and Gobet (2017) found a moderate improvement in children’s memory skills and a small overall improvement in other cognitive domains. Sala and Gobet (2017) highlighted the lack of active controls in past studies as a limitation in determining the true impact of music training. In many schools, music activities with deaf children are popular, especially activities, which include percussion instruments and musical games involving motor skills and rhythm perception.

In sum, deaf children are at risk of delayed EF development, but music-based training could be a way to intervene to improve their EF skills. Music-based training also fits easily into a school day and is an attractive option for many children. The current study evaluated the effectiveness of a music-based EF intervention for deaf children and addressed the following research questions:

1. Does the intervention have a positive effect on deaf children’s EF skills?
2. Are some areas of EF more “trainable” than others?

In order to test the reliability of the intervention, it was used with different groups of children in two studies. This part of the research asked:

1. Can the intervention be replicated in different samples?

We refer to the first instance we ran the music intervention as study 1 and the second occasion as study 2. We first outline the intervention design used across both studies and the tasks used to assess changes in EF, and then describe the participants and results for each study in turn. Then, we discuss both sets of results together in the discussion section.

## Method

Both studies had a within-subjects crossover design, with all participants taking part in both a music-based EF intervention and an art class active control condition. Both conditions consisted of hour-long sessions, twice a week for five weeks (i.e., 10 hours in total for each condition). All sessions were led by an experimenter (the first author), supported by a teaching assistant in each of the schools.

### Intervention Design

For both studies, school-aged children between the ages of 7 and 11 years were recruited and the intervention was designed to be appropriate for this age range. Everyday music activities with deaf school children formed the basis of the intervention, and these were largely rhythm and repetition exercises using percussion instruments. Advice and feedback was given by both deaf and hearing professionals, including two specialist music instructors for deaf children, two teachers of the deaf (TODs), a primary school music teacher, and a special educational needs co-ordinator who was familiar with the use of EF activities with primary school children. See Supplementary data, Supplementary material 1 for more information on EF activities.

### Differentiation and Flexibility within the Intervention

An essential feature of EF interventions is that they should be consistently challenging but not beyond the developmental ability of the child (Diamond, 2012). We did not exclude any children in the study based on intellectual disability. Two broad cognitive ability groupings were made in consultation with teachers. Teachers considered the academic, language, and social abilities of the children in their classrooms and the demands of the intervention. The two-group memberships were corroborated by parents who completed the Behaviour Rating Inventory of Executive Function (BRIEF; Gioia, Isquith, Guy, & Kenworthy, 2000). This is an 86-item questionnaire, which provides detailed information about eight different aspects of children’s EF, arranged into individual scales. Parents are presented with sentences about the child’s behavior such as “*Tries the same approach to a problem over and over even when it does not work*” and is asked to respond by circling N (never), S (sometimes) or O (often). The questions are designed to address children’s ability to initiate behavior, inhibit undesirable responses, demonstrate emotional control, shift attention, monitor progress, plan, and organize themselves and their possessions and use working memory.

Raw scores for the first three scales, (inhibition, shifting, and emotional control) are summed to produce a composite called the “Behavioral Regulation Index (BRI)”. Initiation, working memory, planning, and organization, and monitoring scores are combined to produce a “Metacognition Index (MI)”. The combination of BRI and MI composites provides a “Global Executive Composite (GEC)” for each child. The GEC is an overall summary measure comprising all eight clinical scales mentioned, and is expressed as a *T*-score. A higher *T*-score is indicative of having difficulties with EF, with *T*-scores from 60 to 64 considered to be mildly elevated, and *T*-scores from 65 to 69 considered potentially clinically elevated. *T-*scores at or above 70 are considered clinically elevated. GEC *T*-scores for participants are shown in Table 2.

No children who had been placed in the higher cognitive ability group had any specific EF difficulties reported by their parents. Therefore, low and high cognitive ability versions of each of the activities were developed and thus tailored to children’s needs. See Supplementary data, Supplementary material 2 for differentiated lesson plans.

### Control Activity Design

**Figure 1:**
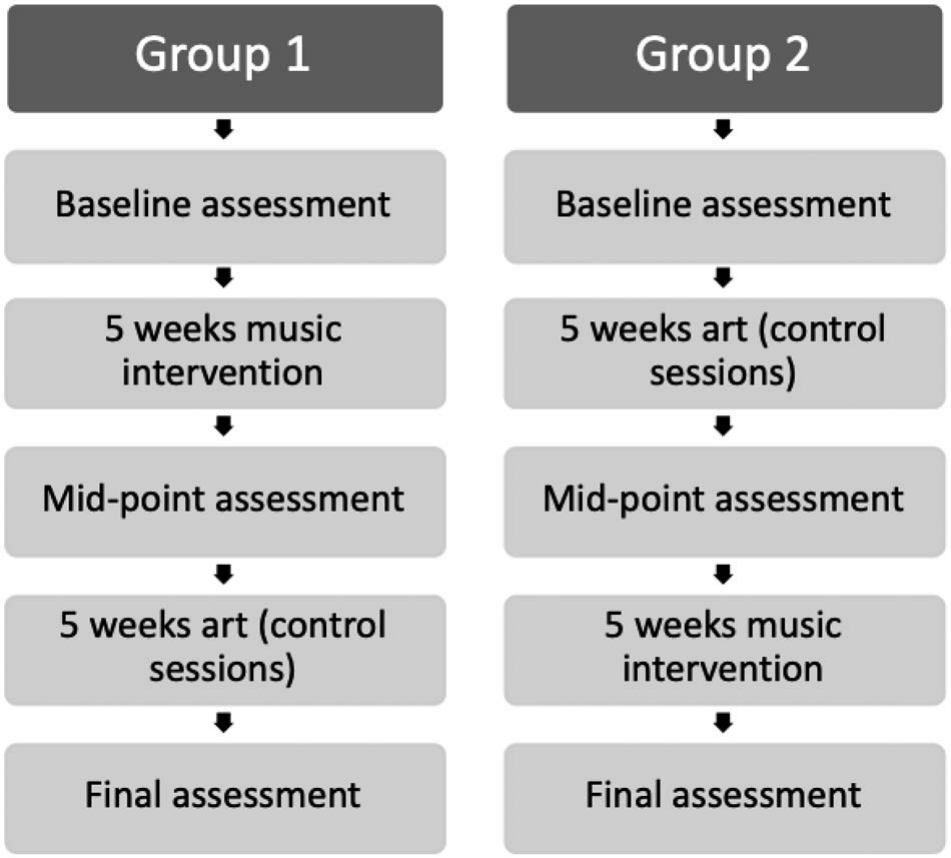
Design of sessions that took place at each school.

The control activity was carefully designed to ensure it involved the same amount of time and adult contact as the EF intervention, but did not specifically focus on or include EF-loaded activities. Previous studies have established art as an appropriate control comparison to music and have not found any EF advantage from these activities (Moreno et al., 2011). To ensure continuity across the art sessions, the theme “the seasons of the year” was chosen. See Supplementary data, Supplementary material 3. This provided two sessions on each season, an additional session where the children produced a rainbow collage and another where they made a themed folder to contain their artwork. There was no need for differentiation for the control activity as teachers judged all sessions were accessible to children of every level of ability; however, each activity needed to be engaging for children between the ages of 7–11 years. The overall design is illustrated in Figure 1.

## Study 1

### Participants

Three schools in England were approached to take part in the intervention. Teachers identified 16 children between the ages of 7–11 years including 5 with additional special needs. The mean age of the participants was 9 years 3 months (SD = 1.17). This is a small but representative sample as it included children of different ability levels and special education needs. We were limited by the availability of children who could commit to several weeks of testing and training. As described previously, the children were divided into groups according to cognitive ability (see Table 1), with seven children in a lower cognitive ability group and nine in a higher group. All three schools were mainstream schools with specialist provision for small numbers of deaf children. Schools A and C have total communication policies with British Sign Language (BSL), Sign Supported English (SSE), and Spoken English being used. The children spend their mornings in the specialist center with a TOD teaching them the core subjects english, literacy and maths, where they receive additional support from deaf and hearing learning support assistants (LSAs). In the afternoons, they attend mainstream classes supported by LSAs and TODs who use both SSE and BSL. The deaf children at School B participate in all mainstream classes and activities. The children are educated orally, however some sign language is occasionally used. A deaf tutor visits the deaf children once a week, providing lessons in sign language and deaf culture.

**Table 1:** Study 1: Information on children’s school, sex, age, hearing aids, special educational needs, language preference, intervention order, ability grouping, and EF composite score

Child code	School	Sex	Age	Aids	SEN	Language preference	Intervention order	Ability group	BRIEF global executive composite *T*-score
1	A	F	9;7	CI	Y	BSL	C-I	Low	81
2	A	M	9;3	BAHA	Y	English + SSE	C-I	Low	72
3	A	M	10;6	CI	Y	BSL	C-I	Low	83
9	C	F	10;11	HA	Y	English + SSE	C-I	Low	54
10	C	M	10;0	CI	Y	BSL	C-I	Low	60
14	C	F	9;1	HA	N	BSL	C-I	Low	55
15	C	M	8;2	CI	N	English + SSE	C-I	Low	44
4	A	M	10;1	HA	N	SSE	I-C	High	53
5	A	F	7;11	HA	N	SSE	I-C	High	38
6	B	M	9;6	HA	N	English	I-C	High	34
7	B	F	8;6	CI	N	English	I-C	High	40
8	B	M	8;6	CI	N	English	I-C	High	36
11	C	M	10;10	HA	N	English + SSE	I-C	High	39
12	C	F	7;2	HA	N	English	I-C	High	45
13	C	M	7;0	HA	N	English	I-C	High	40
16	C	F	10;5	CI	N	English + SSE	I-C	High	46

*BRIEF global executive composite T-scores: T 60–64 are considered mildly elevated; T 65–69 are considered potentially clinically elevated; T > and = 70 are considered clinically elevated*

*Key to table abbreviations*

HA: Hearing aid; CI: cochlear implant; BAHA: bone anchored hearing aid; SEN: special educational need; BSL: British sign language; SSE: sign supported English; I-C: intervention condition followed by control condition; C-I: control condition followed by intervention condition

Due to the wide variation in language levels, preferred method of communication, and educational experiences of deaf children, matching participants with appropriate control participants is extremely difficult. A within-subjects design whereby each child took part in both the music intervention and art (control) sessions was adopted, enabling each child to act as their own control in a crossover design.

In order to fit into the local dynamics of the schools we worked with, there were different patterns of how the intervention and control sessions were run. School B children began with the music intervention for five weeks and concluded with five weeks of art control sessions. At schools A and C, both group sessions were run concurrently. Table 2 also indicates whether children participated in the control condition first, followed by the intervention (C-I), or completed the intervention activities before taking part in the control condition (I-C). As it was necessary for the experimenter to become familiar with individual children’s communication preferences and to build relationships with them, for this study, all children in the lower ability groups took part in the art control sessions first, followed by the music intervention, and all of the children in the higher ability groups completed the music intervention first, before switching to the art sessions.

**Table 2:** Study 1: mean scores and standard deviations on EF tasks by ability group

Task	Ability group	N	Music		Art		Change score (post-test – pre-test)	
			Pre-test (SD)	Post-test (SD)	Pre-test (SD)	Post-test (SD)	Music (SD)	Art (SD)
Visuospatial span score (span size)	High	9	4.56 (1.42)	6.44 (1.74)	6.44 (1.74)	6.11 (1.83)	1.89 (1.69)	−0.33 (1.45)
	Low	7	2.86 (1.68)	4.86 (2.73)	2.29 (1.25)	2.86 (1.68)	2.00 (2.83)	0.57 (1.81)
Odd one out score (number correct)	High	9	7.56 (3.91)	11.22 (2.54)	11.22 (2.54)	10.22 (2.28)	3.67 (2.18)	−1.0 (2.59)
	Low	5	6.80 (2.39)	8.20 (2.39)	5.40 (3.21)	6.80 (2.39)	1.40 (1.34)	1.40 (1.82)
Sun apple task interference^*^ (number correct)	High	9	17.33 (15.32)	11.78 (21.63)	11.78 (21.63)	3.56 (13.79)	−5.55 (27.80)	−8.22 (27.56)
	Low	7	25.11 (24.10)	1.71 (17.58)	21.43 (21.02)	25.11 (24.10)	−14.40 (34.46)	3.68 (2.57)
Tower of London number of moves^*^	High	9	78.44 (17.83)	66.78 (7.58)	66.78 (7.58)	70.22 (8.47)	−11.67 (16.51)	3.44 (13.07)
	Low	6	77.67 (13.05)	68.33 (8.76)	89.33 (23.24)	77.67 (13.05)	−9.34 (7.37)	−11.66 (19.31)
Tower of London time taken in seconds^*^	High	9	288.92 (178.21)	194.06 (64.32)	194.06 (64.32)	153.29 (35.19)	−94.86 (152.62)	−4.77 (64.77)
	Low	6	247.37 (155.77)	186.59 (89.66)	321.03 (199.98)	247.37 (155.77)	−60.78 (75.25)	−73.66 (77.67)
Color trails interference (in seconds^*^)	High	9	1.45 (.42)	1.00 (.37)	1.00 (.37)	0.91 (.46)	−.45 (.49)	0.09 (.53)
	Low	6	1.07 (.61)	1.17 (.54)	0.93 (.61)	1.07 (.60)	0.10 (.92)	0.14 (.82)
Design fluency score (number correct)	High	9	16.00 (6.48)	23.78 (10.15)	23.78 (10.15)	23.78 (7.76)	7.78 (5.89)	0.00 (7.19)
	Low	7	9.43 (2.51)	11.57 (4.12)	9.00 (3.16)	9.43 (2.51)	2.14 (2.43)	0.43 (1.90)

^*^A lower score is indicative of better performance.

### Executive Function Assessments

Children completed the following six non-verbal EF tasks at baseline, post-intervention, and post-control testing times. Testing took approximately one hour, split into four sessions each lasting 15–20mins.

#### Spatial span task

The spatial span task is a measure of visuospatial working memory. Children are presented with an array of 10 blue blocks mounted on a platform in an irregular pattern. They are instructed to tap the blocks in the same order as the experimenter (who is able to see numbers on each of the blocks to aid in the administration of the test). Testing begins with two block strings (with two trials at each level), then increases up to nine block strings, or until the child makes errors in both trials of a particular level. The child’s score consists of the number of correct trials achieved before the task ends or is discontinued.

A second “backwards” condition requires the children to tap the blocks in the reverse order to the examiner (starting with the last block that the examiner tapped) and is scored in the same manner as the “forward” condition. The task begins with two practice trials in both the forward and backward conditions to ensure that the child understands the task. One point is awarded for each trial correctly repeated (Wechsler & Naglieri, 2006).

#### Odd one out task

The odd one out task is a test of executive-loaded visuospatial working memory. Children are presented with three shapes on a power point slide. Two of the shapes are identical, one of them different. Children are asked *“which shape is the odd one out?”* and to point to the different shape. The following slide has a grid with three empty boxes, and the child is asked to point to the location of the previously identified “odd one out” shape.

Complexity is increased after four trials, when children are asked to recall the position of the missing shapes after being presented with two pairs of stimuli on teach trial. After four more trials, complexity increases again to three stimuli to recall, and continues up to a maximum of six stimuli per trial. The test is stopped when children make an error on two (or more) trials in a set. Prior to the start of the test, two practice trials are administered to help the child to understand the task procedure: a single-item and a two-item trial. Correct responses to the practice items are revealed to the child if they do not initially answer correctly. During the test, children are dissuaded from verbalizing to help them remember the location of the shapes (for example, by repeating the location to themselves “right, middle, right,” etc.) and are not allowed to use their hands to mark the location and thus aid their recall (Henry, 2001).

#### Sun apple task

The sun-apple task was administered as a measure of children’s inhibition skills. It is based on the “Simon effect,” which refers to the increased time required to respond to incongruent items (Simon, 1990). The task was presented on a Lenovo laptop and was run using “Presentation” software (Neurobehavioural Systems, Inc., 2013), which controlled the presentation and timing of the stimuli. Stickers are placed on “S” and “K” keys of the laptop keyboard, the left side with a picture representation of an apple and a picture of a sun on the right. Children follow the instructions as they are presented on the screen, or alternatively, the instructions are signed to them. They are told to keep one index finger on the “apple key” and one on the “sun key” and whenever they see a sun or an apple on the screen to press the corresponding key. There are three practice trials to ensure the child understands the task and are able to respond in adequate time (i.e., their responses are neither too slow nor haphazard and fast). The test trials then begin. There are 16 congruent trials (where the apple or sun are presented on the same side of the screen as the response key), and 16 incongruent trials (where the items are presented on the opposite side of the screen to the response key, requiring the children to inhibit incorrect automatic responses and placing a higher load on their EF). The children’s scores on this task consist of their percentage accuracy on both congruent and incongruent scores and their reaction response times to the stimuli. There was an equal number of trials present in all conditions. The data were trimmed and trials where children were too late to respond (i.e., responses timed-out after 900 ms) were removed. Correct responses on EF-loaded incongruent trials were then reported as percentages. An interference score was calculated by subtracting the number of accurate congruent trials from accurate incongruent trials. Interference scores are commonly used in stroop tests and other inhibitory tasks as an accurate measure of a person’s inhibition, based on their baseline accuracy on congruent trials (Simon, 1990).

#### Tower of London task

The Tower of London task was administered on a laptop using Psychology Experiment Building Language (PEBL) Test Battery Software (Mueller & Piper, 2014). This assessment is a traditional problem-solving and planning task, which tests the child’s ability to make and follow plans. It is a task that is regularly included in EF test batteries (Shallice, 1982).

Children are presented with two sets of colored discs, arranged across three columns. The experimenter says to the child—*“Look at the discs with different colors. These discs here [pointing to the top array] belong to the computer. You cannot move them. These discs here [pointing to the lower array] belong to you. You need to make your discs look the same as the computer’s.”* To ensure the child understands how to complete the task, the experimenter assists the child with the first trial (which is subsequently excluded from data analysis). The experimenter tells the child to *“click on the red disc”* and then shows them where to put it*. “Can you see, that is the same as the computer’s? Now, can you make the rest the same by yourself? Try to use as few moves as possible and do it as fast as you can”.* If the child struggles on the first trial, the experimenter is able to assist them and give prompts until they complete the trial. On all subsequent trials, no assistance is given other than encouraging prompts (e.g., *“You are nearly there”*) to encourage the children to keep going. There are eight trials, and achievement is measured by time taken to complete the task, the number of moves taken to complete each trial, and the number of extra moves (i.e., moves made on top of the minimum possible) taken to complete each trial (Shallice, 1982).

#### Color trails task

As a measure of cognitive flexibility, children were given the color trails task. This is a paper and pencil task consisting of two parts. The first task requires the children to connect 15 numbered circles of alternate yellow and pink colors whilst the experimenter times them. This provides a baseline time of the children’s performance on the task. In the second part of the test, they are presented with 30 circles numbered 1–15, 15 of which are yellow and fifteen pink. They are instructed to start on the yellow color and then *“connect it to the next number which is a different color”*. This requires them to remember the rule of switching between colors as they connect circles, and to ignore the distractor circles. The experimenter was careful not to use the words “pink” or “yellow” when giving instructions in accordance with the task protocol. The children are timed on this task, and any color or number errors they make are noted on their score sheet to be included in later analysis. The child’s score on the task consists of the time taken to complete each task, and an interference score is calculated by taking their time to complete the second task from their baseline time on the first task (Llorente, Williams, Satz, & D’Elia, 2003).

#### *Design fluency task (NEPSY-II,*  Korkman, Kirk, & Kemp*, 2007**)*

The design fluency task, taken from the NEPSY-II battery (Korkman et al., 2007), is a pen and paper task designed to measure planning, flexibility and self-monitoring skills. The design fluency task has two conditions. In the first condition children are presented with an array of dots set out in a square structure and are told that they need to create different designs by joining the dots. The experimenter demonstrates the task, emphasizing that they can join as many or as few dots as they please (although it has to be at least two) and that every design they create needs to be different. The experimenter demonstrates two example designs on a practice sheet, and then asks the child to create two more different designs. At this stage, if the child replicates a previous design, they are reminded that every design needs to be different. The child is then presented with an array of 35 boxes of dots, and told to *“draw as many designs as you can, until I tell you to stop”*. The experimenter times the child and instructs them to stop after 1 minute. The child’s score on this task is the number of unique and accurately drawn designs they produce in 1 minute.

## Results

In order to reduce any potential experimenter effects, such as scoring bias, data scoring and analysis began only once data collection had been completed. Scorers were not blind to which participants were in which condition. Children’s average scores on each of the tasks according to ability group are shown in Table 2.

The results for each task are reported as changes in children’s performance on the task at different testing time points. This was calculated from pre- and post-music intervention test scores and pre- and post-art control test scores (Table 3). Comparisons are then made between these two change scores, using a repeated measures t-test (Table 4). Differences in the number of participants on some tasks are due to children not completing the tasks at all three timepoints because of school absences.

**Table 3:** Study 1: mean scores pre and post-test in both conditions, and repeated measures *t*-tests for conditions

Task	*N*	Music		Art		*t*		*p*	*d*
		Pre-test	Post-test	Pre-test	Post-test				
Visuospatial span score (span size)	16	3.81 (1.72)	5.75 (2.29)	4.63 (2.60)	4.69 (2.39)	Music	−3.56	.003^**^	0.96
						Art	−0.16	.879	0.02
Odd one out score (number correct)	14	7.29 (3.54)	10.14 (2.82)	9.14 (3.94)	9.00 (3.01)	Music	−4.91	<.001^***^	0.89
						Art	0.21	.834	0.04
Sun apple task interference (number correct)	16	20.75 (19.33)	11.31 (19.32)	16.00 (21.23)	13.00 (21.35)	Music	1.26	.227	0.49
						Art	0.47	.647	0.14
Tower of London number of moves	15	78.13 (15.58)	67.40 (7.80)	75.80 (18.88)	73.20 (10.77)	Music	3.13	.007^**^	0.87
						Art	0.59	.564	0.17
Tower of London time taken in seconds	15	272.23 (165.10)	191.07 (72.45)	244.85 (144.20)	19.92 (107.94)	Music	2.60	.021^*^	0.64
						Art	3.38	.005^**^	0.42
Color trails interference (in seconds)	15	1.30 (.52)	1.07 (.43)	0.97 (.46)	0.97 (.51)	Music	1.24	.237	0.48
						Art	0.00	1.000	0.00
Design fluency (number correct)	16	13.13 (6.02)	18.44 (10.04)	17.31 (10.78)	17.50 (9.42)	Music	−3.94	.001^**^	0.64
						Art	−.14	.891	0.02

^*^p < .05.

^**^p < .01.

^***^p < .001.

**Table 4:** Study 1: mean change scores for intervention (music) and control (art) conditions and *T*-scores comparing the two conditions

Task	*N*	Change score		*t*	*p*	*d*
		Music	Art			
Visuospatial span score (span size)	16	1.94 (2.17)	0.06 (1.61)	2.22	.042^*^	0.98
Odd one out score (number correct)	14	2.86 (2.18)	−0.14 (2.51)	2.88	.013^*^	1.28
Sun apple task interference (number correct)	16	−9.44 (29.96)	−3.00 (25.70)	−7.87	.443	0.23
Tower of London number of moves	15	−10.73 (13.29)	−2.60 (17.04)	1.44	.172	0.53
Tower of London time taken in seconds	15	−81.23 (121.22)	−53.93 (61.89)	−1.29	.218	0.28
Color trails interference score in seconds	15	−0.23 (.72)	0.00 (.68)	−1.69	.114	0.33
Design fluency score (number correct)	16	5.31 (5.40)	0.18 (5.39)	2.17	.046^*^	0.95

^*^p < .05.

^**^p < .01.

^***^p < .001.

Results demonstrate that children’s scores on the visuospatial span task, odd one out, number of moves on the Tower of London task, and design fluency improved significantly after the music intervention, but not their accuracy scores on the Tower of London, color trails or sun apple tasks. (Note that in the case of the number of moves in the Tower of London task, a lower score indicates fewer moves and therefore a better performance). The art intervention caused no significant changes in score for any of the EF tasks except for the amount of time taken in the Tower of London task. When the change scores for the music and art intervention were directly compared, change scores were significantly higher for the visuospatial span task, odd one out, and design fluency.

## Study 2

Study 2 was carried out to investigate whether the results of study 1 could be replicated in a different set of children, as well as increasing the numbers of participants in the overall intervention. We also wanted to remove the confound between cognitive level and amount of practice on the EF tasks that was present in study 1 (where the lower ability children were all tested in the order control-intervention and therefore had had more practice with the tasks at the post-intervention measurement point compared to the high-ability children, who were all tested in the order intervention-control and who had therefore had less practice with the tasks at the post-intervention measurement point). After considering the results for study 1 and feedback from teachers concerning the time taken to complete the assessments, fewer EF assessments were used in study 2 to reduce time testing while still covering all areas of EF. This reduced the testing time from 60 to 40 minutes. The three main areas of EF (working memory, inhibition and cognitive flexibility) were tested in the spatial span task (visuospatial working memory), the odd one out task (executive-loaded visuospatial working memory), a Simon task (inhibition), and the color trails task (cognitive flexibility).

### Participants

Participants were recruited from two primary schools in England. Both schools are mainstream schools with specialist provision for deaf children. The children attending school D remain in mainstream classes throughout the day, supported by specialist teaching assistants. Children from school E spend the morning at an on-site center focusing on literacy skills, before attending mainstream classes in the afternoon.

Information about the children who took part in study 2 is provided in Table 5, including their sex, age, use of aids (hearing aids or cochlear implants), language preference, and the order in which they took part in the intervention and control conditions. In contrast to study 1, all of the children in study 2 had spoken English as their preferred language, and no children had any additional statement of special educational need. BRIEF questionnaires were not administered in study 2.

**Table 5:** Study 2: information on children’s school, sex, age, hearing aids, language preference, and grouping

Child code	School	Sex	Age	Aids	Language preference	Order	Ability group
5	D	F	6;07	CI	English	C-I	Low
6	D	F	6;09	CI	English	C-I	Low
7	D	M	7;00	CI	English	C-I	Low
9	E	F	9;11	HA	English	C-I	High
10	E	F	10;07	HA	English	C-I	High
11	E	M	10;05	HA	English	C-I	High
12	E	M	5;00	HA	English	I-C	Low
13	E	F	6;04	CI	English	I-C	Low
8	E	F	6;03	CI	English	I-C	Low
1	D	M	7;11	HA	English	I-C	High
2	D	F	8;00	CI	English	I-C	High
3	D	M	7;10	HA	English	I-C	High
4	D	F	8;00	CI	English	I-C	High

### Executive Function Assessments

Because of technical issues, the sun apple task was substituted with a comparable “Simon task,” which is a similar task of inhibitory response but in its running includes a greater number of trials.

### The Simon Task (Inhibition)

This task is a measure of response inhibition, and is presented on a laptop using PEBL software (Mueller & Piper, 2014). In the Simon task, children have to make a rapid judgment of the color of a stimulus while ignoring its horizontal position. A red or blue circle appears on the screen and the children have to respond by pressing the left shift key for a red circle and the right shift key for a blue circle. Colored smiley face stickers were placed over the corresponding shift keys to remind children of the response keys. The task consisted of 140 trials; 70 congruent (where the circle appears on the same side of the screen as the response key) and 70 incongruent (where the circle appears on the opposite side of the screen to the response key). Accuracy of participants’ responses was recorded in an output file. Interference scores were calculated for data analysis by subtracting accurate responses to congruent trials from accurate responses to incongruent trials.

## Results

As with study 1, data scoring and analysis began only once data collection had been completed, in order to reduce any potential experimenter effects or scoring bias. Children’s average scores on each of the tasks according to ability group are shown in Table 6.

**Table 6:** Study 2: mean scores and standard deviations on EF tasks by ability group

Task	Ability group	*N*	Music		Art		Change score (post-test – pre-test)	
			Pre-test (SD)	Post-test (SD)	Pre-test (SD)	Post-test (SD)	Music (SD)	Art (SD)
Visuospatial span (span size)	High	7	9.14 (3.44)	12.29 (2.29)	10.86 (2.34)	12.14 (3.34)	3.15 (1.95)	1.28 (1.70)
	Low	6	8.67 (1.97)	11.33 (1.21)	10.33 (1.75)	9.33 (2.34)	2.67 (1.21)	−1.00 (1.27)
Odd one out (number correct)	High	7	1.43 (2.37)	13.29 (3.04)	11.43 (2.82)	11.00 (2.08)	2.86 (1.35)	−.43 (1.13)
	Low	6	6.83 (1.72)	8.00 (2.10)	6.67 (1.97)	7.83 (1.17)	1.17 (1.17)	1.17 (1.84)
Simon task interference^*^ (number correct)	High	7	−3.14 (2.61)	−0.29 (1.98)	−1.29 (2.43)	−1.14 (2.91)	2.86 (2.80)	.15 (1.11)
	Low	6	−3.83 (2.99)	0.67 (.82)	−1.00 (3.23)	−1.83 (2.32)	4.50 (2.88)	−0.83 (2.14)
Color trails (score in seconds)^*^	High	7	82.14 (29.87)	74.14 (29.27)	79.29 (28.00)	69.71 (21.31)	−8.00 (24.0)	−9.58 (3.74)
	Low	4	81.75 (33.83)	78.00 (29.43)	93.00 (26.77)	79.50 (3.58)	−3.75 (3.50)	−13.50 (6.33)

^*^A smaller number is indicative of better performance.

The changes in children’s performance on the tasks at different testing time points were calculated from pre- and post-music intervention test scores and pre- and post-art control test scores (Table 7). Comparisons were then made between these two change scores, using a repeated measures *t*-test (Table 8) to determine whether the music intervention was effective.

**Table 7:** Study 2: mean scores pre- and post-test in both conditions, and repeated measures *t*-tests for conditions

Task	*N*	Music		Art		*t*		*p*	*d*
		Pre-test	Post-test	Pre-test	Post-test				
Visuospatial span (span size)	13	8.92 (2.75)	11.85 (1.86)	1.62 (2.02)	10.85 (3.16)	Music	−6.57	<.001^***^	1.25
						Art	−.44	.666	0.09
Odd one out (number correct)	13	8.77 (2.74)	10.85 (3.74)	9.23 (3.42)	9.54 (2.33)	Music	−5.00	<.001^***^	0.63
						Art	−.67	.515	0.11
Simon task interference (Number correct)	13	−3.46 (2.70)	0.15 (1.57)	−1.15 (2.70)	−1.46 (2.57)	Music	−4.54	.001^**^	1.63
						Art	0.63	.538	0.12
Color trails score (in seconds)	11	82.00 (29.64)	75.54 (27.88)	84.27 (27.08)	73.27 (24.03)	Music	0.81	.437	0.22
						Art	1.87	.090	0.43

^*^p < .05.

^**^p < .01.

^***^p < .001.

Results show that children’s scores on all tasks except for the color trails task increased significantly after the music intervention. The art intervention led to no significant changes in score for any of the EF tasks. When the change scores for the music and art intervention were directly compared, changes scores were significantly higher after the music intervention for all tasks, with the exception of color trails.

## Discussion

The first research question asked: Does the music-based intervention have a positive effect on deaf children’s EF skills? The results revealed improvement in deaf children’s visuospatial/executive-loaded visuospatial working memory. Significant improvements were also found in post-intervention design fluency scores (a measure of flexibility and planning) in study 1. In the second study inhibitory skills also improved. This is the first study to find that EFs can be improved in deaf children. This finding is strengthened by the inclusion of an active control condition and replication in two separate samples. It is also promising that improvements were found following a relatively short intervention of 10-hours duration. The possibility of an EF intervention being successful is important, considering the negative impact poor EF has on a range of outcomes for deaf children (Botting et al., 2017; Figueras et al., 2008; Hall et al., 2016; Jones et al., 2020; Morgan & Dye, 2020). These abilities are an important part of children’s wider success in controlling and regulating social and emotional skills (Andersson, 2008; Blair & Razza, 2007; Thorell, Lindqvist, Bergman Nutley, Bohlin, & Klingberg, 2009). At the same time, we did not find large improvements, nor across all EFs, for all deaf children.

**Table 8:** Study 2: mean change scores for intervention (music) and control (art) conditions and *T*-scores comparing the two conditions

Task	*N*	Change score		*t*	*p*	*d*
		Music	Art			
Visuospatial span (span size)	13	2.92 (1.61)	0.23 (1.88)	4.06	.002^**^	1.12
Odd one out (number correct)	13	2.25 (1.42)	0.08 (1.51)	3.07	.011^*^	0.67
Simon task interference (number correct)	13	3.62 (2.84)	−0.31 (1.75)	3.67	.003^**^	1.01
Color trails accuracy score (in seconds)	11	−6.45 (26.46)	−11.00 (19.46)	−0.74	.478	0.22

^*^p < .05.

^**^p < .01.

^***^p < .001.

Our second research question asked: Are some areas of EF more “trainable” than others? The pattern of results across different EFs and individual children was complex. Considering the overall results, working memory and inhibition emerge as two EFs amenable to training. In both studies 1 and 2, no effect of intervention was found for the color trails task (cognitive flexibility). However, patterns within the data for different cognitive ability groups suggest that within working memory and inhibition, the difficulty level of the task is important for achieving training effects (see Tables 4 and 8). This complexity is related to the point that EF training at the child’s level of potential development is the most effective at improving EF skill (Diamond, 2012). The deaf children in the training studies were heterogeneous. Within the sample, there were children whose general cognitive abilities were considered by their teachers to be typical and others whose cognitive abilities were considered to be lower. Results across both studies indicate that children in the lower cognitive ability group saw the most significant improvement to their inhibitory skills post-intervention in comparison to post-control performance. For working memory, in study 1, deaf children in the lower ability group saw improvements to their visuospatial working memory post-intervention, but not on the more difficult odd one out task.

The pattern of results also links to the premise that inhibition is one of the core EFs that is likely to underpin other EFs such as planning and fluency (Miyake & Friedman, 2012). In the current study, children in the lower ability group showed the most improvement in inhibitory skill after a period of training. The higher cognitive ability group showed less improvement in inhibition. Little effect of the intervention is seen for the lower ability group on planning fluency. Conversely, the significant improvement in the design fluency scores at post-intervention compared to post-control, appears to be driven by improvements in the higher cognitive ability group (see Tables 4 and 8). Our complex findings on ability level demonstrate the importance of considering individual needs, strengths, weaknesses, and abilities when implementing EF interventions and training.

The final research question asked: Can the intervention be replicated in different samples? We observed improvements to EF in both studies, which suggests the intervention was robust enough to transfer to other samples. Replication entailed a range of school settings and across a heterogeneous group of deaf children using different languages and communication systems. We did not find large effect sizes across all EFs and for all deaf children. Both studies were small scale and included children of different abilities. The inclusion of a wide range of children in both studies strengthens the ecological validity of the intervention, and its suitability for real-world classroom environments. This is positive in terms of how representative the sample is but can make any consistent group effects difficult to attain.

There are some limitations to our study that we raise here. Any research design carries strengths and weaknesses. We chose a crossover design because matching participants with appropriate control participants is extremely difficult with deaf children due to the high heterogeneity. Our design enabled each child to act as their own control in a crossover design. However, crossover designs are less powerful in producing lasting impact on the outcome of interest. There is also the limitation imposed by the necessary test–retest methodology of assessment (Chan, Shum, Toulopoulou, & Chen, 2008). The improvements made by children on some of the tasks over time may represent expected practice effects as a function of increased familiarity with the tasks, and this was a particular limitation of study 1 where ability group and intervention order were conflated, meaning that the low-ability group had more practice with the task at the post-intervention testing point. As a result, it is possible that the effectiveness of the intervention for the low-ability group was over-estimated and, conversely, its effectiveness for the high-ability group under-estimated. This limitation was addressed by the way we allocated children in study 2, where we decoupled ability grouping from intervention order. Future work in this area might instead beneficially use a single-subject design. Single-subject designs are frequently used in intervention studies, which involve atypical populations, as they provide a powerful tool for determining the effects of different treatment conditions (Lee Swanson & Sachse-Lee, 2000, for a meta-analysis of single-subject interventions). Such designs are less vulnerable to the concerns about practice effects that were a limitation of our study 1, and are more suitable for interventions that are expected to have a lasting impact.

While we observed the EF intervention had a positive impact, by looking at EF performance in the group that had the control then intervention activity we see these effects were not maintained once intervention was completed. There is the possibility that our crossover design requiring time between conditions to allow consolidation before a control condition phase. Future replications of our intervention might usefully include a period of consolidation between conditions. Additionally, the short-term effects we observed suggest the importance of continued EF intervention in deaf children so as to achieve sustained results. It is assumed, longer interventions will produce more robust effects (Bowmer et al., 2018; Diamond & Lee, 2011). Thus, future replications could consider more than a 5-week intervention period.

In conclusion, working memory and inhibition can be improved through short time-scale, classroom-based musical training. Future studies should include more time between intervention and control conditions, as well as, post intervention in order to evaluate maintenance. Related to varying cognitive abilities of deaf children, our research shows improvements after training were not uniform across all participants. The study highlights the importance of supporting the development of EF skills alongside or in tandem with any therapy work around speech and language in educational settings.

## Supplementary Material

EFtrainingSupplementary_material_1_enab026

EFtrainingSupplementary_2_enab026

EFtrainingSupplementary_3_enab026
