# Supplementary material for: Executive Function Training for Deaf Children: Impact of a Music Intervention
Source: J Deaf Stud Deaf Educ. 2021 Sep 2;26(4):490–500. doi: 10.1093/deafed/enab026 (PMC8448422; doi:10.1093/deafed/enab026)
Supplement: EFtrainingSupplementary_material_1_enab026 [file eftrainingsupplementary_material_1_enab026.docx]

Supplementary material 1. The EF intervention activities and their associated areas of EF

| Activity | Description | Associated area of |
| --- | --- | --- |
| Follow the Leader | Children have to copy whatever the ‘leader’ does while the leader changes their actions frequently. One child who is not taking part has to guess who the leader is. Therefore, transitions need to be subtle and participating children need to pay attention and adapt to the new action quickly. All children take turns as the leader and the guesser. | Requires planning from the leader and monitoring/attention/ inhibition from the participants. |
| The Body Orchestra | Children learn to associate pictures of different animals to corresponding actions and sounds. The game requires working memory as children need to remember which action accompanies which animal and instrument. | This activity introduces composition, which requires planning skills and working memory |
| The Naughty/Good Orchestra | Children need to attend to the changing instructions of the conductor  i.e. if they should play fast/slow, loud/quiet.  The Naughty orchestra requires inhibition and working memory skills, as children are required to do the opposite of what they are instructed to do. The conductor can give directions in quick succession to increase the difficulty of the game. | Inhibition |
| Making our own Music | Introducing children to basic musical notation enables them to create simple rhythms for others to play. These sessions can be easily differentiated using colours and symbols instead of music notation. | Memory, planning |
| Switch! | A rhythmic version of the “N-Back”, whereby children have to remember the previous rhythm and hold it in memory. E.g. seated, start by clapping a simple rhythm on your knees, then start clapping your hands. When you start clapping your hands, the children should start clapping on their knees. Then you tap your shoulders, and the children should start clapping their hands etc. Adults in the room can model the game to help make the rules clear. | Working memory, monitoring, attention, inhibition |
