# Supplementary material for: Executive Function Training for Deaf Children: Impact of a Music Intervention
Source: J Deaf Stud Deaf Educ. 2021 Sep 2;26(4):490–500. doi: 10.1093/deafed/enab026 (PMC8448422; doi:10.1093/deafed/enab026)
Supplement: EFtrainingSupplementary_2_enab026 [file eftrainingsupplementary_2_enab026.doc]

Supplementary material 2. Full lesson plans with differentiated activities

Successful interventions for executive functions require a lot of repetition and practice of skills. For that reason, many of the activities in this programme are repeated over the course of the 10 sessions.

It is anticipated that some of the children may find particular activities harder than others, therefore each activity has the potential for differentiation according to ability. Even though the lesson plans should be followed, some flexibility must be given to account for individual differences between the children, and the teacher needs to make sure that each child is able to grasp and complete the activity before moving on to a more challenging version. However, it is equally important that the children are continuously challenged by the programme, and are encouraged to keep attempting tasks that they may find difficult, with adults demonstrating and scaffolding techniques when appropriate.

Motivation is also an important factor and some of the games include a competitive element (such as ‘don’t clap this one back’, where the motivation is for the class to collectively beat the teacher over the course of the 10 sessions).

If children appear too frustrated or bored with any of the activities, the activity can be swapped to another on the programme that they prefer. This is working on the basis that motivation and interest will be more beneficial, as all of the activities are designed to incorporate the training of similar areas of EF.

Two games will be used to open and close every session:

1. **“Pass the Rhythm game”** – will be at the start of every session. Sat in a circle, the teacher claps out a rhythm and passes it on to the next student in the circle until it has passed all the way around the circle. Continue playing and increasing the difficulty of the rhythm until there is only one child left – the winner!
2. **“Don’t clap this one back”** – every session ends with a game of don’t clap this one back, standing up in a circle. The teacher claps a range of rhythms, no more than 4 beats long, using hands, body, and the floor and the children copy the rhythms. However, if the teacher claps the rhythm 'Don't clap this one back' – (
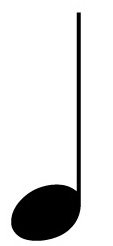

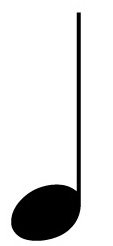

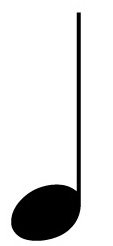

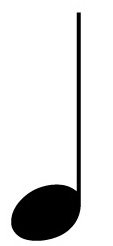

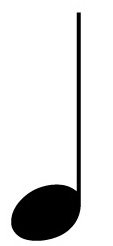
) they don't clap it back. Every time someone claps the rhythm when they aren't supposed to, the teacher gets a point. If no one claps when it’s 'don't clap this one back' the children get a point. Who will win at the end of the term, children or the teacher? This provides motivation for the children to play the game and pay attention.

| Follow the Leader | Children have to copy whatever the ‘leader’ does while the leader changes their actions frequently. One child who is not taking part has to guess who the leader is. Therefore, transitions need to be subtle and participating children need to pay attention and adapt to the new action quickly! All take turns as the leader and the guesser. Requires planning from the leader and monitoring/attention/ inhibition from the participants. |
| --- | --- |
| The Body Orchestra | Attending to different timbres and textures. The game requires working memory as children need to remember which action accompanies which animal and instrument. This session introduces composition which requires planning skills |
| The Naughty/Good Orchestra | Children need to attend to the changing instructions of the conductor. The Naughty orchestra requires inhibition and working memory skills, as children are required to do the opposite of what they are instructed to do. The conductor can give directions in quick succession to increase the difficulty of the game. |
| Making our own Music | Introducing children to basic musical notation enables them to create simple rhythms for others to play. This requires planning and working memory. These sessions can be easily differentiated. |
| The memory game | A working memory game requiring children to recall as many items as they can from a tray containing 20 music-related items. |
| Switch! | A rhythmic version of the “N-Back”, this game requires working memory, monitoring and attention. |
| The Rhythm Machine | Different simple rhythms played by each child require inhibition and selective attention skills. Children are encouraged to attend to their own playing, and then the group as a while whist maintaining their rhythm. This requires monitoring and flexibility. |

**Week 1 Session 1 – Drums and Rhythms**

| **Executive Function Focus**  Working memory, attention and switching | |
| --- | --- |
| **VOCABULARY**  Conductor  Rhythm  Baton | **RESOURCES**  Drums – ideally one each  Baton /special conductor’s hat  Powerpoint |
| **INTRODUCTION**  Introductions (Introduce self and what we will be doing 2 lessons every week)  “Hello/welcome” song  Short video of different drums and where they come from (Japan, Africa, Orchestral)  **ACTIVITY**  GAME 1 – Copying. First demonstrate different rhythms to the children. Can include clapping.  Go around each person having a turn to play a rhythm for 20 seconds. All others watch carefully and copy (Adults assist if any children struggle). This is where the game “pass the rhythm” can be introduced. Explain the rules to the children and play a simple version of the game.  Game 2 – “Follow the Leader”. One child leaves the room. A leader is chosen to play a rhythm and will change it occasionally. Played without drums, just clapping and stamping feet. Child enters the room and has to work out who the ‘leader’ is.  Game 3 – The Conductor.  Demonstrate conducting – use signs to reinforce concepts faster, slower, louder, quieter. Practice these on the drums  Children take turns being the conductor. When the conductor points to you, you play your drum and pay attention to their requests …. Faster, quieter, louder etc. Conductor can point to individual players or to everyone.  **PLENARY**  Short overview of what we did today.  “Goodbye” song/ Introduce “Don’t clap this one back” game  Tidying up. | |
| **Differentiation**   - Continue the copying game instead of “follow the leader” - Conducting - limit to single concept e.g. fast/slow | |

**Week 1 Session 2 – Body Orchestra**

| **Executive Function Focus**  Working memory, switching. | |
| --- | --- |
| **VOCABULARY**  Texture  Timbre | **RESOURCES**  Shakers,  One drum,  Clappers/woodblock  2-3 laminated sets of “body orchestra” cards  Powerpoint |
| **INTRODUCTION**  “Hello/welcome” song. Pass the rhythm game  Introduce today’s topic – Body orchestra  Focus on different timbre and texture of instruments.  **ACTIVITY**  Activity 1 – Using powerpoint and allowing the children to handle the instruments, talk about different sounds the instruments make. Use “body orchestra” flash cards in the powerpoint.  Activity 2 – Once the link between the instrument, animal, and body movement have been established, children can take turns being the ‘conductor’, The conductor hands out animal cards to the other students and arranges how they like, in a line. They will then conduct the body orchestra!  When it is the turn of the next conductor, different cards will be handed to the children and they have to remember the body action that corresponds with it.  Activity 3 – In groups, play the memory card game using the “body orchestra” cards. When a child picks up a pair, they have to produce the corresponding action.  **PLENARY**  Overview of what we did today  “Goodbye” song/ Don’t clap this one back  Tidy up | |
| **Differentiation**   - Link instrument and body movement (leave out animals) - Simplified version of the memory card game with fewer cards. | |

**Week 2 ~ Session 3 – Making Our Own Music**

| **Executive Function Focus**  Planning, attention, flexibility | |
| --- | --- |
| **VOCABULARY**  Rhythm  Tempo | **RESOURCES**  Four Chairs  Simple percussion (tambourines/shakers)  Blue and red coloured sheets of paper |
| **INTRODUCTION**  Hello song. Pass the Rhythm game  What are we doing today? – Composing our own music (focus on rhythm and tempo)  **ACTIVITY**  Game 1 – “Quick and slow” introduce children to simple and basic concepts of notation and time value of notes.  Use colour codes for value of notes. Red for crotchet, blue for a quaver. Practice clapping examples of 4/4 lines of music.  Create a visual ‘notation tree’. “Tea, Coffee”  Game 2 – composing your piece.  Working in pairs, children use coloured paper to compose their own rhythms. After 10 mins, come back to circle and present their compositions…. Everyone claps/uses percussion to play the rhythms.  Game 3 – People composition!  Four chairs at the font and six volunteers. Demonstrate how to compose using people. If sat on chair normally = crotchet, if sat to the side = half a crotchet (quaver) note. Ie. Two people sat back to back followed by one sat normally can be clapped as – *quick quick slow.*  Children take turns to physically move people into position to create their own rhythm. Everyone plays the rhythm. Can re-introduce the concept of loud and quiet from last session to make it more interesting, e.g. if standing = loud, if seated = quiet.  **PLENARY**  Come back to the circle  Overview of what we did today  Goodbye song/ Don’t clap this one back game  Tidy up | |
| **Differentiation**   - Use alternative simple symbols for notation - Focus on game 1 & 2 only | |

**Week 2 ~ Session 4 The Memory Game and Naughty Orchestra**

| **Executive Function Focus**  Working memory, inhibition and attention | |
| --- | --- |
| **VOCABULARY**  Vocabulary linked to percussion instruments and musical items depending on what is sourced. | **RESOURCES**  20 music related items/percussion.  Tray  Cloth to cover all the items  Paper and pencils  Small prize (or prizes in case of a draw)  Baton / special conductor’s hat |
| **INTRODUCTION**  Hello/welcome song. Pass the rhythm game  Introduce today’s topic – learning about new instruments  **ACTIVITY**  Activity 1 – Memory game. Being with the tray of items covered with the cloth. Children sit in a semi-circle around the tray. Produce each time one at a time, giving its name and how it is played/works then return it underneath the cloth before removing the next one.  Explain the memory game to the children. Remove the cloth and give them 1min to memorise as many items as they can. After 2 mins, cover the items again and encourage the children to write down as many items as they can remember – there is a small prize for whoever can remember the most. Adults can support those children needing help writing/spelling and items can be signed.  Activity 2 – Explore the instruments. Children have 10mins to play with the instruments and see how many different ways they can use them. Instruments are passed around. Children do not need to remain seated for this part.  Activity 3 – The “Naughty Orchestra”. Using the items from under the cloth and building on the “orchestra” game from a previous session. All the children choose an instrument and play the Orchestra game. After 3 children have had a go as conductor, the rules will change. This time the children have to do the OPPOSITE of what the conductor requests – session leader to model as the “naughty orchestra” player first.  After a few children have had a go conducting, the naughty orchestra, alternate between the “good” and “naughty” orchestra.  **PLENARY**  Overview of what we did today  Goodbye song/ don’t clap this one back game  Tidy up | |
| **Differentiation**   - Activity 3 - practice following conductor. Only move on to “naughty orchestra” if able to, to increase challenge. | |

**Week 3 ~ Session 5 – Switch!**

| **Executive Function Focus**  Working memory, attention, inhibition | |
| --- | --- |
| **VOCABULARY**  Switch | **RESOURCES**  Drums  Shakers,  Clappers/woodblock  2-3 laminated sets of “body orchestra” cards  Powerpoint |
| **INTRODUCTION**  Hello song/ pass the rhythm game  **ACTIVITY**  Activity 1 – Switch!! Begin with a game of “follow the leader” to remind children of the rules.  After a couple of rounds, introduce the harder “switch” element. Children continue the rhythm while the leader changes their action. When the leader says “switch!” (visual cue) the children have to copy the previous action whilst the leader performs a new one. Keep the rhythms and actions simple. Musical version of an N-Back. Eg. seated, start by clapping a simple rhythm on your knees, then start clapping your hands. When you start clapping your hands, the children should start clapping on their knees. Then you tap your shoulders, and the children should start clapping their hands etc. Adults in the room can model the game to help make the rules clear.  When this activity is repeated in later sessions, difficulty can be increased by having to remember actions that were 2-back.  Activity 2 – Body orchestra. Re-visit the game where children take turns assigning the cards and conducting the orchestra. When the children successfully remember all of the movement/animals/instruments, introduce some more cards to the pack.  They can then split into pairs and use the cards to play the memory card game.  **PLENARY**  Overview of what we did today  Goodbye song / don’t clap this one back game  Tidy up | |
| **Differentiation**   - Continue follow the leader game instead of Switch activity . Introduce more variations such as leader playing loud/quiet, fast/slow. | |

**Week 3 ~ Session 6 – Making Our Own Music**

| **Executive Function Focus**  Planning, attention, flexibility | |
| --- | --- |
| **VOCABULARY**  Rhythm  Tempo | **RESOURCES**  Four Chairs  Simple percussion (tambourines/shakers)  Blue, red , yellow and green coloured sheets of paper  Drums if available |
| **INTRODUCTION**  **ACTIVITY**  Activity 1 – Revisit “Quick and slow” introduce children to simple and basic concepts of notation and time value of notes.  Use colour codes for value of notes. Red for crotchet, blue for a quaver. Practice clapping examples of 4/4 lines of music.  ***Providing the children mastered this in the last session, introduce semi quavers and minims on yellow and green paper. Those who find it harder, stick with crotchets and quavers. This time, the notation can be written on the back of the paper.***  Create a visual ‘notation tree’. – “Tea, Coffee”  Activity 2 – composing your piece.  Working in pairs, children use coloured paper to compose their own rhythms. After 10 mins, come back to circle and present their compositions. Everyone claps/uses percussion to play the rhythms.  Activity 3 – The Rhythm Machine.  Using simple rhythms that the children just created, one child starts by playing their rhythm over and over again on the floor. The person sitting to the left of the starter then adds their own rhythm to go along with the rhythms that are already going. Again, the rhythm has to repeat and can not change.  When everyone has added a rhythm, let the children listen for a few measures.  The first person will then stop doing their rhythm. Again, pause for a second or two, and then the second person drops out.  It continues until the last person is the only rhythm.  Encourage the children to attend to the changes in sound and how one part can make a HUGE difference.  **PLENARY**  Overview of what we did today  Goodbye song / don’t clap this one back game  Tidy up | |
| **Differentiation**   - Instead of activity 3, continue activity 2 with variations of tempo and loud/quiet. Also vary instruments if available | |

**Week 4 ~ Session 7 – Naughty or Good Orchestra**

| **Executive Function Focus**  Working memory, inhibition and attention | |
| --- | --- |
| **VOCABULARY**  Vocabulary linked to percussion instruments and musical items depending on what is sourced. | **RESOURCES**  20 music related items/percussion.  Tray  Cloth to cover all the items  Paper and pencils  Small prize (or prizes in case of a draw)  Baton / conductor’s special hat |
| **INTRODUCTION**  Hello song/pass the rhythm game.  What are we doing today? – Memory Game and Good or Naughty orchestra.  **ACTIVITY**  Activity 1 – The memory game. Repeat the memory game as in the previous session but introducing items one by one and covering them up with a cloth. This time, use 10 items that appeared in the previous session, and 10 new items. This time, there is an added bonus for the person who can remember the most items, and also which were on the tray before and which are new.  Activity 2 - The naughty orchestra**.** All the children choose an instrument from the tray and play the Orchestra game. After 3 children have had a go as conductor, play the naughty orchestra.  After a few children have had a go conducting, the naughty orchestra, alternate between the “good” and “naughty” orchestra – the child conducting can decide what they want the orchestra to be, good or naughty  **PLENARY**  Overview of what we did today  Goodbye song / don’t clap this one back game  Tidy up | |
| **Differentiation**   - Develop skills from previous sessions. Attempt “naughty orchestra” | |

**Week 4 ~ Session 8 – Switch!**

| **Executive Function Focus**  Working memory, attention, inhibition | |
| --- | --- |
| **VOCABULARY (optional)**  Switch  Crotchet  Quaver  Semi quaver  Minim | **RESOURCES**  Coloured sheets of paper  Notation posters/ rhythms  Examples of 4/4 bars for children to play using quavers, crotchets, minims and semiquavers |
| **INTRODUCTION**  Hello song/ pass the rhythm game  What are we doing today? – Switch and making our own music  **ACTIVITY**  Activity 1 – Switch!! Begin with a game of “follow the leader” to remind children of the rules, but this time, nobody leaves the room. Only copying.  After a couple of rounds, introduce the harder “switch” element. Children continue the rhythm while the leader changes their beat. When the leader says “switch!” (visual cue) the children have to copy the previous beat whilst the leader performs a new one. Keep the rhythms simple. ***This time try introducing 2-back. Children can practice this in pairs, while the teacher goes around and individually plays “switch” with each child in order to see how each of them copes with the task.***  Activity 2 – Making our own music. Continue with exploring notation and beating different rhythms (preparation for next session, where simple syncopation is introduced). This activity needs to be differentiated according to the abilities of each child. More advanced can move on from coloured paper to simple notation.  Activity 3 – People composition! Children are given cards with simple rhythm notation on them. They then have to re-create the rhythm using people (as they did in session 3) and then clap the rhythm when they are done. All children to check whether they have got it right.  **PLENARY**  Overview of what we did today  Goodbye song / don’t clap this one back game  Tidy up | |
| **Differentiation**   - Revisiting activities from previous sessions - introduce “switch” element for some children who may be able to do it. Possibly do this 1:1 if additional adult support is available. | |

**Week 5 ~ Session 9 – Rhythmic Patterns 1**

| **Executive Function Focus**  Working memory, inhibition, attention, planning | |
| --- | --- |
| **VOCABULARY**  Metronome | **RESOURCES**  Drum for each child  Printed copies of simple rhythms  Printed copies of syncopated rhythms  Metronome with flashing light  Posters with review of basic music note vales and examples of syncopated rhythms |
| **INTRODUCTION**  Hello song/ pass a rhythm  What are we doing today –  **ACTIVITY**  Activity 1 – Switch!! Begin with a game of “follow the leader” to remind children of the rules, but his time, nobody leaves the room. Only copying.  After a couple of rounds, introduce the harder “switch” element. Children continue the rhythm while the leader changes their beat. When the leader says “switch!” (visual cue) the children have to copy the previous beat whilst the leader performs a new one. Keep the rhythms simple. ***This time try introducing 2-back. Children can practice this in pairs, while the teacher goes around and individually plays “switch” with each child in order to see how each of them copes with the task.***  Activity 2 – Making our own music. Continue with exploring notation and beating different rhythms (preparation for next session, where simple syncopation is introduced). This activity needs to be differentiated according to the abilities of each child. More advanced can move on from coloured paper to simple notation.  Activity 3 – People composition! Children are given cards with simple rhythm notation on them. They then have to re-create the rhythm using people (as they did in session 3) and then clap the rhythm when they are done. All children to check whether they have got it right.  **PLENARY**  Overview of what we did today  Goodbye song / don’t clap this one back game (try and make the scores close in the penultimate session!)  Tidy up | |
| **Differentiation**   - Instead of activity 3, continue with “naughty orchestra” | |

**Week 5 ~ Session 10 – Rhythm Patterns 2**

| **Executive Function Focus**  Working memory, inhibition, attention, planning | |
| --- | --- |
| **VOCABULARY**  Metronome | **RESOURCES**  Drum for each child  Printed copies of simple rhythms  Metronome with flashing light  All percussion and instruments from previous sessions |
| **INTRODUCTION**  Hello song/ Pass the rhythm  What are we doing today? – last music session  **ACTIVITY**  Activity 1 – Continue with the more complicated rhythms from the previous session. Follow the same procedure, re-capping notation values and beating out the rhythms together first.  Activity 2 – Children are allowed to select their favourite instrument from the previous sessions and explain what they like about it. What different sounds can they make with it? How is it different from other instruments? This can be followed by a game of Nice/naughty orchestra with the selected instruments.  Activity 3 – Final game of follow the leader.  **PLENARY**  Overview of what we did today  Feedback from the children – did they enjoy the music sessions? Were they fun, easy, hard, boring?? What did they enjoy and what didn’t they like?  Goodbye song / don’t clap this one back game – for the final time! Look at the score board who is going to win? Children or teacher?  Tidy up | |
| **Differentiation**   - N/A | |
