# Supplementary material for: Executive Function Training for Deaf Children: Impact of a Music Intervention
Source: J Deaf Stud Deaf Educ. 2021 Sep 2;26(4):490–500. doi: 10.1093/deafed/enab026 (PMC8448422; doi:10.1093/deafed/enab026)
Supplement: EFtrainingSupplementary_3_enab026 [file eftrainingsupplementary_3_enab026.docx]

Supplementary material 3. Full lesson plans for control activity sessions

The activities detailed in the following lesson plans form the active control condition for a study investigating the effect on executive function skills of a music-based intervention for deaf children. The art activities described here are designed to have as little demand on children’s executive function skills as possible. Therefore, children are presented with an example of the intended finished art work, prior to beginning the activities and they are guided by adults who help them complete the activities if necessary. As they receive support in the activities and they are designed to be as easy as possible for the children, no specific differentiated tasks have been devised.

The theme of the art activities is “Seasons of the year”. There are 10 sessions – 8 of which cover the 4 seasons of the year (i.e., 2 sessions are spent on each season), one session where the children make a rainbow collage, and one session where they make a folder in which to keep all of the artwork they produce over the course of the study. If children appear too frustrated or bored with any of the activities, they can be given the option of colouring in pre-printed scenes based on the appropriate season. If children don’t complete their activity in a particular session, they may complete it at the end of the next session if there is time.

**Week 1 Session 1 – Rainbows**

| **RESOURCES**  Coloured tissue paper Coloured paint Felt pens and colouring pencils  Coloured felt squares Glitter paints  Scissors Newspaper to cover work surfaces  White A3 paper Coloured buttons  Rainbow print out and picture examples |
| --- |
| **INTRODUCTION**  Introduce the topic to the children. Ask them about the seasons of the year – what are they? What kinds of colours are associated with each season? What season is it right now? Over the next 5 weeks we will be making art work related to the seasons of the year. Today we are starting with a fun activity… making rainbow collages.  **ACTIVITY**  Show the children example pictures of rainbow collages.  Give each child a piece of A3 white paper. They can choose to draw their rainbow free-hand or use the print out as a template.  Children have choice of using paint, colour pencils, glitter paint, felt, tissue paper and/or coloured buttons on their collage.  **PLENARY**  Short overview of what we did today, everyone shows their work.  Tidying up. |
| **EVALUATION/NOTES ON THE SESSION** |

**Week 1 Session 2 – Making Folders for Art Work**

| **RESOURCES**  A3 Coloured card Seasons of the year cover page  Hole punch Glue  Treasury tags  Colouring-in print outs |
| --- |
| **INTRODUCTION**  Show the children an example of the folder they will make  **ACTIVITY**  Let them choose which colour paper they want to use for their folder – 2 pieces  Hole punch both pieces for the children and attach treasury tags  Children glue the cover page to the front and decorate the folders with their colouring pictures  **PLENARY**  Overview of what we did today  Tidy up |
| **EVALUATION/NOTES ON THE SESSION** |

**Week 2 ~ Session 3 – Winter (1) Snowflakes**

| **RESOURCES**  Snowflake templates Glue  Scissors Paint brushes  Glitter paint Newspaper to cover work surfaces  A4 paper |
| --- |
| **INTRODUCTION**  What are we doing today? Introduce ‘Winter’ topic of the week. What colours do we associate with winter? Show the children the silver and blue glitter paints. Show them examples of paper cut-out snowflakes already made.  **ACTIVITY**  Give each of the children a template of the snowflakes. Show them how to fold the paper and cut out their own snowflakes. They can make their own if they wish.  Snowflakes are then stuck to A4 white paper and the children can decorate them with blue and silver glitter paints.  **PLENARY**  Overview of what we did today  Tidy up |
| **EVALUATION/NOTES ON THE SESSION** |

**Week 2 ~ Session 4 – Winter (2) Penguins**

| **RESOURCES**  Black Card Scissors  White A3 paper Glue  Orange Card glitter paint  Stick on “Googly eyes” |
| --- |
| **INTRODUCTION**  Introduce today’s topic – second session of winter. Making penguins. Show the children an example of a pre-made penguin picture.  **ACTIVITY**  Children create the penguins by layering white paper on top of the black paper (shapes can be pre-cut for children who would have difficulty cutting out)  Children cut an orange triangle for the beak and add eyes to the penguins  The finished penguins are then mounted on white paper which can be decorated and painted with glitter/paint.  **PLENARY**  Overview of what we did today  Tidy up |
| **EVALUATION/NOTES ON THE SESSION** |

**Week 3 ~ Session 5 – Spring (1) – Blossom Trees**

| **RESOURCES**  Light and dark pink, green and white tissue paper  Felt tip pens  Glue Print outs of tree trunk template  Paint brushes  Colouring pencils |
| --- |
| **INTRODUCTION**  Talk about springtime – what colours are associated with spring? etc. Show children an example of blossom tree art they are about to create.  **ACTIVITY**  Children can choose to use the print out of a tree trunk to begin their picture, or draw their own tree trunk using pencils and felt tip pens.  The children tear up pieces of the tissue paper and screw them up into a ball. These are then glued to the paper to represent the leaves and blossoms on the tree.  **PLENARY**  Overview of what we did today  Tidy up |
| **EVALUATION/NOTES ON SESSION** |

**Week 3 ~ Session 6 – Spring 2 – Spring scene and collage**

| **RESOURCES**  Tissue paper in spring colours (yellow, pink, light green) A4 white paper  Paints – various colours Spring coloured buttons and feathers  Colouring pencils and felt tip pens  Scissors Spring scene print outs  Glue |
| --- |
| **INTRODUCTION**  Show children the different spring scene print outs and allow them to choose 2 different ones.  **ACTIVITY**  Children can create a spring collage by cutting out the spring scenes and decorate using paint, colouring pencils and tissue paper, coloured buttons and feathers.  **PLENARY**  Overview of what we did today  Tidy up |
| **EVALUATION/NOTES ON SESSION** |

**Week 4 ~ Session 7 – Summer (1) – Holiday colouring scenes**

| **RESOURCES**  Tissue paper in bright colours A4 white paper  Paints – various colours  Colouring pencils and felt tip pens  Scissors Holiday scene print outs (beach, park, fishing)  Glue |
| --- |
| **INTRODUCTION**  Show children the different summer scene print outs and allow them to choose 2 different ones.  **ACTIVITY**  Children can create a spring collage by cutting out the summer scenes and decorate using paint, colouring pencils and tissue paper,  **PLENARY**  Overview of what we did today  Tidy up |
| **EVALUATION/NOTES ON SESSION** |

**Week 4 ~ Session 8 – Summer (2) – Butterfly symmetry**

| **RESOURCES**  A4 white paper  Various coloured paints  Paintbrushes  Water pots Newspaper to cover work surfaces |
| --- |
| **INTRODUCTION**  What are we doing today? Show children examples of painted butterfly symmetry pictures. Demonstrate how to make the butterfly by folding the paper in half and making a wing design on one side of. Fold the other half over and press firmly to produce a symmetrical wing on the other side. Fill in the butterfly body in the middle.  **ACTIVITY**  Give each child a piece of A4 white paper and help them when needed in completing the task. Children can make as many butterflies as they wish. Cut paper into smaller sections to produce various size butterflies. Cut them out once dry.  **PLENARY**  Overview of what we did today  Tidy up |
| **EVALUATION/NOTES ON SESSION** |

**Week 5 ~ Session 9 – Autumn (1) – Hedgehogs**

| **RESOURCES**  A4 white paper  Glue (optional: leaves collected from playground)  Scissors  Hedgehog print out Felt tip pens |
| --- |
| **INTRODUCTION**  What are we doing today – Introduce Autumn. What colours do we need etc. Show children a completed picture of hedgehog collage  **ACTIVITY**  Children each take a print out of the hedgehog outline to colour in and mount on a piece of paper. They then use leaves, colours pens and any other materials they choose to represent the hedgehog spikes. Then they decorate/colour in the background however they wish using appropriate autumnal colours.  **PLENARY**  Overview of what we did today  Tidy up |
| **EVALUATION/NOTES ON SESSION** |

**Week 5 ~ Session 10 – Autumn (2) – Squirrel/Autumn collage**

| **RESOURCES A4 white paper**  Glue  Scissors Brown/yellow/ grey feathers  Squirrel print out Felt tip pens |
| --- |
| **INTRODUCTION**  What are we doing today? – last art lesson together. Children have the opportunity to finish off any uncompleted work and put everything in their folders to take home.  **ACTIVITY**  Children each take a print out of the squirrel outline to colour in and mount on a piece of paper. They then use feathers, colours pens and any other materials they choose to represent the squirrel’s tail. Then they decorate/colour in the background however they wish using appropriate autumnal colours.  **PLENARY**  Overview of what we did today  Feedback from the children – did they enjoy the art sessions? Were they fun, easy, hard, boring?? What did they enjoy and what didn’t they like?  Tidy up  Put all artwork into folders to take home to their parents |
| **EVALUATION/NOTES ON SESSION** |
